# Noninvasive Detection of Smooth Muscle Cell-Derived Hot Spots to Study Atherosclerosis by PET/MRI in Mice

**DOI:** 10.1161/CIRCRESAHA.122.322296

**Published:** 2023-02-16

**Authors:** Susanne Feil, Dimitri Stowbur, Barbara F. Schörg, Walter Ehrlichmann, Gerald Reischl, Manfred Kneilling, Bernd J. Pichler, Robert Feil

**Affiliations:** Interfakultäres Institut für Biochemie (S.F., R.F.), University of Tübingen, Germany.; Department of Preclinical Imaging and Radiopharmacy, Werner Siemens Imaging Center (D.S., B.F.S., W.E., G.R., M.K., B.J.P.), University of Tübingen, Germany.; Cluster of Excellence iFIT (EXC 2180) “Image-Guided and Functionally Instructed Tumor Therapies” (D.S., G.R., M.K., B.J.P.), University of Tübingen, Germany.; Department of Dermatology (M.K.), University of Tübingen, Germany.; German Cancer Consortium and German Cancer Research Center, Heidelberg, Germany (B.J.P.).

**Keywords:** magnetic resonance imaging, mice transgenic, myocytes, smooth muscle, positron-emission tomography

In atherosclerosis, a thick smooth muscle cell (SMC)–rich fibrous cap stabilizes plaques and, thus, reduces the risk of heart attack or stroke. SMCs show tremendous plasticity and can switch from contractile to modulated plaque cells with diverse phenotypes.^1,2^ Interestingly, modulated SMCs show clonal growth, so that distinct cell patches or hot spots in a plaque are presumably derived from only few parental cells. It is not yet clear whether the modulated SMCs play a disease-promoting or protective role in atherogenesis. This is at least, in part, due to the lack of tools to selectively visualize SMC-derived plaque cells in vivo in a living animal. Here, we describe a novel Cre/lox-based approach for noninvasive detection of SMC hot spots in advanced atherosclerotic plaques of mice using positron emission tomography (PET) combined with magnetic resonance imaging (MRI).

For in vivo imaging experiments, we crossed the tamoxifen-inducible SM22-CreER^T2^ mouse line^3,4^ to the Cre-switchable sr38tk PET reporter mouse line^5^ on an atherosclerotic ApoE (apolipoprotein E)-deficient background. After tamoxifen induction, these animals express the sr39 variant of herpes simplex virus thymidine kinase (sr39tk) stably and specifically in SM22+ SMCs and their progeny including modulated SMCs that may have lost expression of SMC marker proteins. The PET tracer [^18^F]FHBG (fluoro-3-[hydroxymethyl]butyl)guanine) accumulates in sr39tk-expressing cells after sr39tk-mediated phosphorylation, enabling noninvasive in vivo detection of SMC-derived plaque cells by PET. To activate sr39tk expression in SMCs before the onset of atherosclerosis, 6- to 8-week-old male and female mice were treated with tamoxifen once daily for 5 consecutive days. [^18^F]FHBG PET imaging studies were performed in 12- to 18-month-old mice that had received normal chow and developed advanced atherosclerotic plaques (+athero). Nonatherosclerotic PET reporter mice (−athero) and animals without the PET reporter (no sr39tk) were used as controls. MRI scans were performed for anatomical coregistration. [^18^F]FHBG uptake was independent of sex and negligible in mice lacking the sr39tk PET reporter enzyme (Figure [A], left; Figure [B and D]). Importantly, noninvasive PET/MRI detected clear hot spots of tracer uptake in the aortic arch of atherosclerotic reporter mice (Figure [A], middle; blue circled region) but not in nonatherosclerotic reporter animals (Figure [A], right). Considering that we used a sparse labeling strategy,^4^ the hot spots most likely reflect clonally grown patches of SMC-derived cells. Note that our mouse model visualizes distinct regions of advanced lesions but not all plaques present in our experimental mice. Additional PET signals detected in the lung and salivary gland (Figure [A]) were presumably due to the high number of SM22+ SMCs/myoepithelial cells in these tissues and did not interfere with imaging of the aorta. Quantification of the PET signals recorded in vivo in the aortic arch showed significantly increased tracer uptake in atherosclerotic versus nonatherosclerotic aortae (1.08±0.19 versus 0.61±0.06 %ID/mL; n=5 animals; mean±SEM), while tracer uptake in other analyzed organs was independent of atherosclerosis (Figure [B]). To confirm that the strong in vivo PET signals were derived from plaque SMCs, we dissected the aortae of the scanned animals and subjected them to extensive ex vivo analyses. Visualization of [^18^F]FHBG by autoradiography showed that tracer uptake indeed colocalized with plaque-rich regions detected by bright-field microscopy (Figure [C]) and also confirmed the presence of tracer hot spots in the plaque (Figure [C], left; dark spots). Biodistribution analysis of the tracer indicated a trend for increased uptake in the whole aorta of atherosclerotic versus nonatherosclerotic reporter animals (Figure [D]). The atherosclerotic aorta that showed hot spots of tracer uptake (Figure [A], middle; Figure [C], left) was further analyzed by immunostaining of serial plaque sections for the SMC marker, SMA (smooth muscle alpha-actin), and for a marker of modulated SMCs, MAC2^2,4^ (also known as galectin-3 or LGALS3). As shown in Figure [E], SMA was predominantly expressed in the media and fibrous cap, while the core region contained MAC2+/SMA− cells. This staining pattern indicated that the hot spots detected by PET indeed represent a combination of SMA+ SMCs and large patches of SMC-derived MAC2+ modulated plaque cells. The presence of SMC-derived cell clusters in the plaques of our mouse model was confirmed with reporter mice expressing lacZ (stained blue with X-Gal; Figure [F], left) or an RFP (red fluorescent protein; Ai14 mice; Figure [F], right; Figure [G]) in SMCs instead of the sr39tk reporter. In RFP reporter mice, we could clearly detect SMC-derived cells (red) in the plaque core that were SMA− and MAC2+ (Figure [G]).

**Figure.**
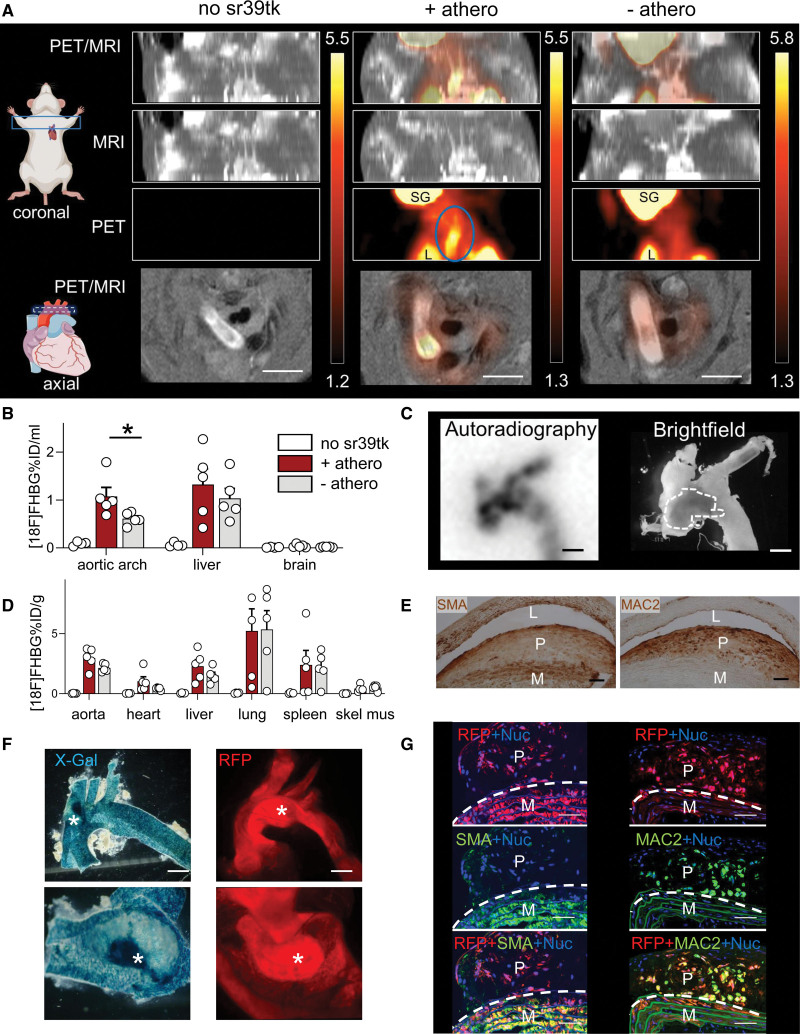
**In vivo positron emission tomography (PET)/magnetic resonance imaging (MRI) and ex vivo analysis of atherosclerosis in SMC-specific reporter mice. A**, Representative [^18^F]FHBG (fluoro-3-[hydroxymethyl]butyl)guanine) PET images of control animals (no sr39tk; **left**) and SMC-specific reporter mice expressing sr39tk (the sr39 variant of the herpes simplex virus thymidine kinase) with (+athero; **middle**) and without atherosclerosis (−athero; **right**). To confirm uptake into distinct anatomic locations, MR images were recorded and overlaid with PET images (PET/MRI). Values of [^18^F]FHBG uptake (in Bq/mL) are indicated by a color gradient. The blue circled region shows a plaque containing a potential SMC-derived hot spot. **B**, The in vivo [^18^F]FHBG uptake was quantified by determination of an equal circular volume of interest (VOI; see representative blue circle in **A**) in the aortic arch or other tissues. PET signals were normalized to injected tracer dose (ID) and VOI volume (in %ID/mL). Data points represent individual animals (n=4–5 per group, mean±SEM). Tracer uptake in VOIs of tissues from atherosclerotic and nonatherosclerotic mice was compared using the Mann–Whitney *U* test (nonparametric). **C**, Representative [^18^F]FHBG autoradiograph and bright-field image of the aortic arch analyzed in **A**, **middle**. **D**, Ex vivo analysis of [^18^F]FHBG biodistribution in the whole aorta and other tissues (in %ID/g) isolated from the same animals as shown in **B** (n=4–5 per group, mean±SEM). **E**, Serial sections of the plaque region with high tracer uptake shown in **A** and **C** were immunostained for SMA (smooth muscle alpha-actin) or MAC2 (galectin-3). **F**, Images of the aortic arch of SMC-specific reporter mice expressing lacZ (stained blue with X-Gal; **left**) or RFP (red fluorescent protein; **right**). **G**, Representative sections of advanced atherosclerotic plaques of SMC-specific RFP reporter mice immunostained for SMA or MAC2. The dashed line marks the border between media and plaque core. Scale bars: 3 mm (**A**), 1 mm (**C** and **F**), and 50 µm (**E** and **G**). L indicates lumen (in **E**); L, lung (in **A**); M, media; Nuc, nuclei; P, plaque region; and SG, salivary gland; skel mus, skeletal muscle; X-Gal, 5-Brom-4-chlor-3-indoxyl-β-D-galactopyranosid. *P=3.2×10^−2 in **B**; *SMC-derived cell patches in the whole mount (**top**) or opened aortic arch (**bottom**) in **F**.

Collectively, our data indicate that PET/MRI in SMC-specific sr39tk reporter mice enables noninvasive quantitative detection of clonally grown SMC-derived cell clusters in atherosclerotic plaques in vivo and possibly many other pathologically altered tissues, such as aneurysms or fibrotic tissues. In contrast to the widely used bacterial artificial chromosome (BAC) transgenic Myh11-CreER^T2^ mouse line, which only allows analysis of male mice (https://www.jax.org/strain/019079), the Cre mouse line used in the present study expresses CreER^T2^ under control of the endogenous SM22 promoter and allows to study both male and female mice. In the future, the SMC-specific PET reporter mice should be useful for longitudinal tracking of SMCs at different time points in the same animal. These studies will help to determine the behavior, function, and therapeutic potential of SMCs in vivo during the progression of atherosclerosis and other diseases with an SMC component.

## Data Availability

Animals were generated by crossing SM22-CreERT2 (Tagln<tm1(cre/ERT2)Feil>) with ApoE null mice (B6.129P2-Apoe<tm1Unc>) and sr39tk (B6;129 Gt(ROSA)26Sor<tm3(ACTB-tdTomato,-sr39tk)Feil>) or lacZ B6-Gt(ROSA)26Sor<tm1Sor> or Ai14 (B6-Gt(ROSA)26Sor<tm14(CAG-tdTomato)Hze>) animals. Male and female experimental mice with the following genotypes were injected intraperitoneally with tamoxifen for 5 consecutive days with a daily dose of 1 mg: SM22-CreERT2^wt/Cre^; ApoE^KO/KO^; sr39tk^wt/flox^ (+athero); SM22-CreERT2^wt/Cre^; sr39tk^wt/flox^ (−athero); SM22-CreERT2^wt/Cre^, ApoE^KO/KO^; sr39tk^wt/wt^ (no sr39tk); SM22-CreERT2^wt/Cre^; ApoE^KO/KO^; lacZ^wt/lacZ^; SM22-CreERT2^wt/Cre^; ApoE^KO/KO^; Ai14^wt/Ai14^. Experiments were approved by the local authority (Regierungspräsidium Tübingen, IB 01/13, IB 07/18G). The methods, data, and materials are available upon request.

## Article Information

### Sources of Funding

This work was supported by grants from the EU Framework Programme for Research and Innovation “Horizon 2020”–ERA-CVD JTC2017-044, the Deutsche Forschungsgemeinschaft (German Research Foundation) Projektnummer 335549539–GRK 2381 and Projektnummer 374031971–TRR 240, and the Dr K.H. Eberle Foundation.

### Disclosures

None.
